# Investigation of Retinal Microcirculation in Diabetic Patients Using Adaptive Optics Ophthalmoscopy and Optical Coherence Angiography

**DOI:** 10.1155/2022/1516668

**Published:** 2022-01-19

**Authors:** Florian Baltă, Irina-Elena Cristescu, Andrada-Elena Mirescu, George Baltă, Mihail Zemba, Ioana Teodora Tofolean

**Affiliations:** ^1^“Carol Davila” University of Medicine and Pharmacy, 050474 Bucharest, Romania; ^2^Clinical Emergency Eye Hospital, 030167 Bucharest, Romania; ^3^“Retina” Clinic, 014142 Bucharest, Romania; ^4^Manchester Royal Eye Hospital, Manchester University Hospitals NHS Foundation Trust, Manchester, UK; ^5^Department of Ophthalmology, “Ovidius” University of Medicine, 900470 Constanta, Romania; ^6^Department of Ophthalmology, “Dr. Carol Davila” Central Military Emergency University Hospital, 010825 Bucharest, Romania

## Abstract

The current research approaches the retinal microvasculature of healthy volunteers (17 subjects), patients with diabetes mellitus without retinopathy (19 subjects), and of diabetic patients with nonproliferative (17 subjects) and proliferative (21 subjects) diabetic retinopathy, by using adaptive optics ophthalmoscopy and optical coherence ophthalmoscopy angiography. For each imaging technique, several vascular parameters have been calculated in order to achieve a comparative analysis of these imaging biomarkers between the four studied groups. The results suggest that diabetic patients with or without diabetic retinopathy prove signs of retinal arteriole structural alterations, mainly showed by altered values of wall to lumen ratio, calculated for the superior or inferior temporal branch of the central retinal artery, near the optic nerve head, and significant changes of the vascular density in the retinal superficial capillary plexus. Both adaptive optics ophthalmoscopy and optical coherence ophthalmoscopy angiography are providing useful information about the retinal microvasculature from early onset of diabetic disease, having a promising diagnostic and prognostic role in the future.

## 1. Introduction

Retina is, regarding its metabolism, one of the most active tissues in the human body, due to increased consumption of oxygen and nutrients. Nowadays, there are more and more available data about the pathogenesis of complex retinal diseases [1–3]. Under normal conditions, the ocular system adapts in order to ensure the retinal metabolic demands, thus providing an appropriate visual function.

Several pathologies, such as diabetes mellitus (DM) [2], arterial hypertension, and other cardiovascular disorders [3], target the vessels, determining morphological and functional changes, which can be essentially mirrored by the status of the retinal vessels.

One of the leading causes of vision loss and blindness worldwide is diabetic retinopathy (DR), a microvascular complication of DM [2]. The Early Treatment Diabetic Retinopathy Study (ETDRS) considers the vascular lesions in the retinal layers to be the hallmark of DR [4, 5]. Furthermore, several papers have shown that among the first changes spotted in the retina of diabetic patients are the ones in the vascular calibres [6–8]. Therefore, retinal vessel parameters may become valuable biomarkers for diabetic retinopathy [8].

In order to structurally and functionally assess a microvascular network, both the topology and the geometric abnormalities are to be investigated [9]. The retinal microcirculation has a vascular pattern similar to a fractal, each part providing similar characteristics with the main structure. A decreased fractal dimension has been shown to be associated with diabetic retinopathy [5, 10]. In order to appreciate the geometry of a vascular network, both the length and the diameters of the vessels are of great interest, in order to calculate different derived parameters.

The current research approaches the retinal microvasculature of healthy volunteers, patients with DM without DR and of diabetic patients with nonproliferative and proliferative diabetic retinopathy, by using adaptive optics ophthalmoscopy (AOO) and optical coherence tomography angiography (OCT-A). These two noninvasive techniques are both powerful and exhibit complementary information regarding the retinal vascular network. On one hand, AOO focuses on the arteriolar structure, while OCT-A delivers high-resolution volumetric angiography images, using motion contrast in seconds. For each imaging technique, several vascular parameters have been calculated in order to achieve a comparative analysis of these imaging biomarkers between the four studied groups.

Adaptive optics ophthalmoscopy is a modern device that uses a cutting-edge technique allowing the visualisation and assessment of the retinal microvasculature, in a noninvasive manner [11]. When using AOO, the retinal arterioles lumen appears to have a bright reflex, while the walls correspond to the darker neighbouring regions. The wall thickness of the blood vessels depends on vessels' size, with large lumens requiring thicker walls. The ratio between the wall thickness and the lumen diameter is known as the wall to lumen ratio (WLR). Among other vascular parameters, WLR has been shown to be significantly correlated with the severity of the diabetic retinopathy [12].

In the recent years, optical coherence tomography angiography has become a valuable tool for diabetic patients, competing with the gold standard fluorescein angiography by allowing the noninvasive quantification of several retinal vascular parameters (vessel density, vascular length density, vessel diameter index, area of the foveal avascular zone, etc.). These quantitative measures may be able to detect changes with the severity and progress of DR.

## 2. Materials and Methods

### 2.1. Study Participants

The current research includes seventy-four eyes from seventy-four patients consulted in the Retina Clinic, Bucharest, in 2019 and 2020. According to the ETDRS guidelines [13], the study population was divided into 4 groups: healthy volunteers (17 eyes), subjects diagnosed with diabetes mellitus without retinopathy (19 eyes), diabetic patients with nonproliferative retinopathy (NPDR) (17 eyes), and diabetic patients with proliferative retinopathy (PDR) (21 eyes).

All the participants were adult Caucasians (>18 years old), with small refractive errors (less than 3 spherical diopters; less than 2.5 cylindrical diopters), without significant ocular media opacities or medical history (except for DM and diabetic retinopathy related procedures).

### 2.2. Subjects' Examination

All participants were conducted a complete ophthalmologic evaluation, namely, medical history, best corrected visual acuity (BCVA), intraocular pressure (IOP), and slit lamp examination of the anterior and posterior segments. In order to better visualise the eye fundus, topical administration of Phenylephrine 10% and Tropicamide 1% was used in patients with relatively small pupillary diameters (<4.5 mm).

rtx1™ adaptive optics retinal camera (Imagine Eyes, Orsay, France) and a swept-source OCT device (DRI OCT Triton, TOPCON Inc., Tokyo, Japan) were both used for microcirculation assessment. Optical biometry (Aladdin, TOPCON Inc., Tokyo, Japan) was used in order to measure the axial length of the included eyes.

### 2.3. Image Processing

**Figure 1:**
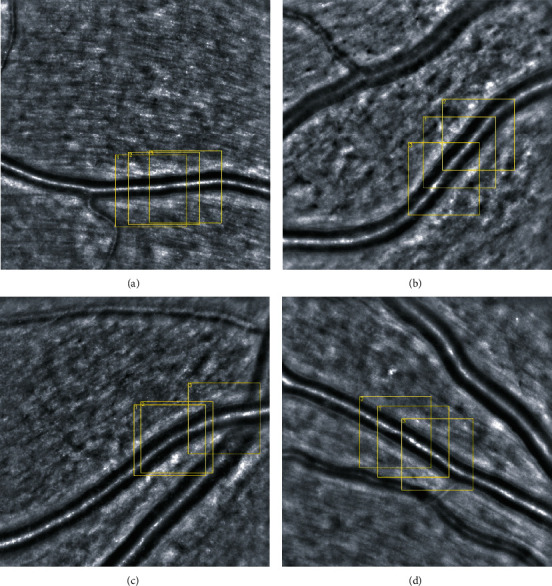
Superior/inferior temporal branch of the central retinal artery—adaptive optics ophthalmoscopy image. (a) Healthy volunteer. (b) Diabetic patient without retinopathy. (c) Diabetic patient with nonproliferative retinopathy. (d) Diabetic patient with proliferative retinopathy. Employing AOdetect software, the mean wall to lumen ratio was calculated from the three selected regions of interest, for each time landmark (100 *μ*m width and height each).

Adaptive optics images were analysed using manufacturer's software (AO detect artery, Imagine Eyes, France) that automatically generates the following vascular parameters for the selected regions of interest: vessel diameter (VD), lumen diameter (LD), mean wall thickness (WT), wall to lumen ratio (WLR), and cross sectional area of the vascular wall (WCSA) (Figure 1).

**Figure 2:**
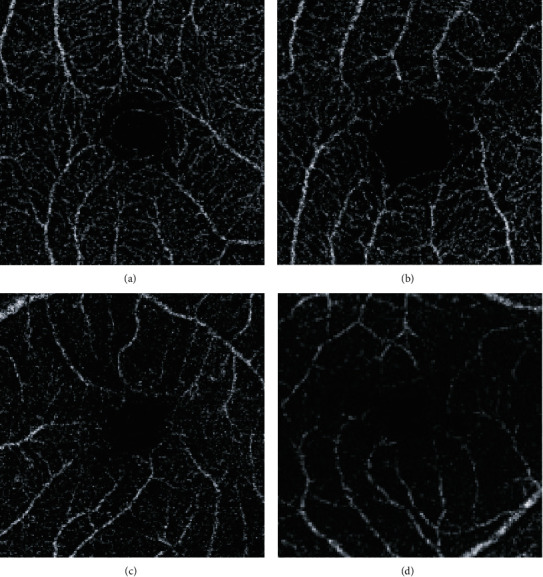
DRI OCT Triton OCT-A 3 × 3 mm*en face* images for the superficial capillary plexus. (a) Healthy volunteer. (b) Diabetic patient without retinopathy. (c) Diabetic patient with nonproliferative retinopathy. (d) Diabetic patient with proliferative retinopathy.

The OCT instrument software (IMAGEnet® 6, TOPCON Inc.) provided, after automated layer segmentation and projection artefacts exclusion, 3 × 3 mm*en face* images for the superficial retinal layer (from 2.6 *μ*m below the internal limiting membrane to 15.6 *μ*m below the interface of the inner plexiform layer and inner nuclear layer) (Figure 2).

**Figure 3:**
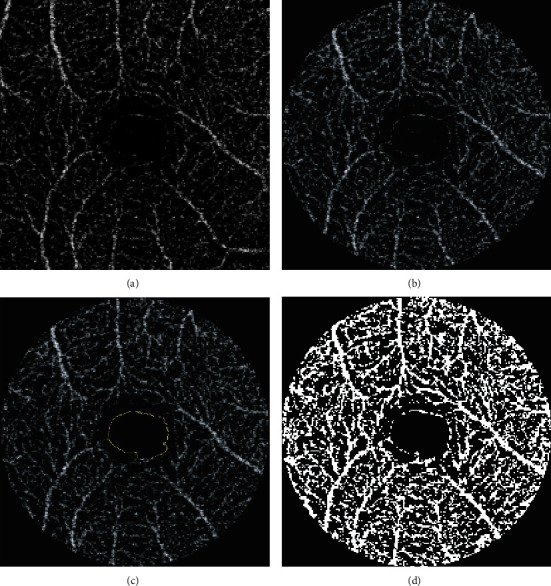
(a) DRI OCT Triton OCT-A 3 × 3 mm*en face* image of the superficial retinal layer in a healthy volunteer. (b) Previous image was cropped to a central circle with 3 mm diameter, using ImageJ software. (c) Manual FAZ area measurement (seen in yellow) in the OCT-A section, using ImageJ software. (d) The *en face* image from (c), transformed into a 8-bit grey scale picture and binarised using an automated thresholding algorithm (mean) from ImageJ software, in order to calculate the vascular density in the superficial capillary plexus.

ImageJ software (Version 1.8; National Institutes of Health, Bethesda, MD, USA) was used in order to calculate the OCT-A related vascular parameters: vascular density along the superficial capillary plexus (SPD), as well as the foveal avascular zone area (FAZ) measured in the superficial capillary plexus (Figure 3). Vascular density is, by definition, the percentage represented by vessels in a selected area [14]. In order to be calculated, the *en face* images were first transformed into 8-bit grey scale pictures, which were further binarised using an automated thresholding algorithm (mean). FAZ was manually sketched and further adjusted for axial length [12].

### 2.4. Statistical Analysis

IBM SPSS Statistics software (Version 23; Armonk, NY: IBM Corp) was used in order to perform descriptive statistics on the collected data. All the results are expressed as mean ± standard deviation. *p* values under 0.05 are considered statistically significant.

Shapiro-Wilk's was the test of choice for normality assessment. Groups' characteristics were compared applying the Kruskal Wallis test or one-way ANOVA analysis. Vessels' parameters were compared between the four groups using one-way ANOVA test, followed by Tukey (for data with equal variances) or Games-Howell (for data with unequal variances) post hoc analysis. Moreover, one-way ANCOVA was conducted having age as a covariate, followed by post hoc analysis with a Bonferroni adjustment. A Spearman's rank-order correlation was applied to determine whether there were any correlations between BCVA and the vascular parameters.

## 3. Results and Discussion

74 eyes from 57 diabetic patients (36 males and 21 females; 47 subjects with type II DM and 10 subjects with type I DM) and 17 healthy volunteers (8 males and 9 females) were enrolled in the current study (Table 1). 26 diabetic patients are insulin-dependent. The mean age of all the study participants was 52.81 ± 15.13 years, while the mean duration of diabetes mellitus in the three groups with diabetic subjects was 9.73 ± 9.67 years. There were significant differences between the controls and each diabetic group (no DR, NPDR, and PDR groups, respectively) considering the age (*p* = 0.018, 0.009, and 0.016, respectively). Significant differences were also found between the number of patients with diabetic maculopathy in the no DR group and NPDR (*p* < 0.001) and PDR group (*p* = 0.009), respectively. BCVA was significantly lower in the RDP group when compared to the no DR (*p* = 0.003) and control group (*p* < 0.001). The control group had a significant higher BCVA than the NPDR group (*p* = 0.002).

21 out of 38 eyes with diabetic retinopathy have associated maculopathy, 19 eyes underwent panretinal photocoagulation (PRP), 22 patients were injected anti-VEGF molecules, and 4 patients underwent pars plana vitrectomy (PPV), all ophthalmic interventions dating more than 6 months before the inclusion into the current study.

There were no significant differences between the groups concerning the values of VD (*F* (3, 37.487) = 1.684, *p* = 0.187), LD (*F* (3, 36.899) = 1.142, *p* = 0.345), and WCSA (*F* (3, 73) = 2.739, *p* = 0.050). WLR was found to have a significant variation (*F* (3, 37.065) = 5.992, *p* = 0.002) between the groups, according to the one-way Welch ANOVA test. Post hoc Games-Howell analysis showed that in the control group WLR had smaller values (0.24 ± 0.046) compared to the no DR group (0.30 ± 0.073, mean difference = −0.059, 95%CI = −0.113, −0.004; *p* = 0.029) and PDR group (0.34 ± 0.10, mean difference = −0.0959, 95%CI = −0.166, −0.025; *p* = 0.005) values (Tables 2 and 3).

Having age as a covariate, the statistical analysis found no significant differences between the groups concerning the values of VD (*F* (3, 69) = 2.029, *p* = 0.118), LD (*F* (3, 69) = 1.837, *p* = 0.148), and WCSA (*F* (3, 69) = 2.156, *p* = 0.101). WLR was found to have a significant variation (*F* (3, 69) = 3.587, *p* = 0.018) between the groups, according to the ANCOVA analysis. Post hoc analysis showed that in the control group, WLR had smaller values compared to the PDR group values (mean difference = −0.085, 95%CI = −0.161, −0.008; *p* = 0.022) (Table 4). Having examined our vascular parameters between male and female groups, only the “superficial capillary density” has shown statistically significant differences. Running the statistics having the duration of diabetes as a covariate, out of all parameters, significant differences were found for wall-to-lumen ratio only.

With respect to the OCT angiography parameters, the FAZ values (*F* (3, 63) = 1.587, *p* = 0.202) did not vary significantly between the groups. The SPD values decreased with the advance of retinopathy (*F* (3, 63) = 202.61, *p* = 0.001). The control group (34.18 ± 1.40) had higher SPD values than the NPDR (32.27 ± 1.93) (mean difference = 1.909, 95%CI = 0.436, 3.382; *p* = 0.006) and the PDR group (32.31 ± 1.61, mean difference = 1.866, 95%CI = 0.393, 3.339; *p* = 0.007). In addition to this, the PDR group had a significantly lower SPD compared to the no DR group (33.85 ± 1.40, mean difference = 1.537, 95%CI = 0.017, 3.05; *p* = 0.046) (Tables 2 and 3).

Having age as a covariate, FAZ values (*F* (3, 58) = 0.545, *p* = 0.653, *F* (3, 59) = 0.763, *p* = 0.519, *F* (3, 59) = 1.026, *p* = 0.388) still did not vary significantly between the groups, while SPD values exhibited similar changes (*F* (3, 59) = 5.608, *p* = 0.002). The control group had higher SPD values than both the PDR group (mean difference = 1.712, 95%CI = 0.010, 3.414; *p* = 0.048) and the NPDR group (mean difference = 1.840, 95%CI = 0.253, 3.427; *p* = 0.015, age as covariate) (Tables 2 and 4).

A Spearman's rank-order correlation was run to assess the relationship between BCVA and AO vascular and OCT-A parameters in diabetic patients. There was a statistically significant, strong negative correlation between the best corrected visual acuity and wall thickness (*r*_*s*_ (57) = −0.367, *p* = 0.005) and wall to lumen ratio (*r*_*s*_ (57) = −0.438, *p* = 0.001), respectively (Table 5).

Further statistical analysis was focused on both AO and OCT-A parameters, comparing subjects that received anti-VEGF injections/retinal laser treatment/vitrectomy prior to the inclusion in this study with untreated participants. Significant differences were found only for the superficial capillary density parameter only between the diabetic patients that received anti-VEGF injections and those that did not (*p* = 0.007). Wall thickness (*p* = 0.011) and WCSA parameters (*p* = 0.043) varied significantly between the subjects with and without diabetic maculopathy.

## 4. Conclusions

Retinal circulation should be analyzed from different perspectives. Larger branches (like the superior/inferior branches of the central retinal artery) are easily assessed using AO-related parameters. Retinal microcirculation can be further examined looking at macular capillary plexuses, which are broadly studied with OCT-A and exhibit functional, topographical, and anatomical particularities. A third “window” to the retinal circulation is the analysis of the microvasculature changes in the peripheral retina, which represents an uncovered topic by the current research.

In this control study, focused on subjects without or with different stages of diabetic retinopathy, we assessed the retinal arterioles using the rtx1 adaptive optics retinal camera. By using a principle taken from astronomy, the device allows a detailed noninvasive visualisation of retinal structures at a histological resolution [14]. In addition to this, OCT angiography was performed in order to describe the macular retinal capillaries plexus flow densities and FAZ areas. According to our hypothesis, proportional vascular changes can be tracked in different stages of diabetic retinopathy, slight alterations being present even before any documentable clinical sign.

Our results suggest that diabetic patients with or without diabetic retinopathy prove signs of retinal arterioles and capillaries structural alterations, as shown by the WLR and SPD parameters. The study groups excluded subjects with untreated arterial hypertension or other vascular disorders. Furthermore, any disease-related intervention, such as retinal laser treatments, vitrectomy, or intravitreal drug injections, were performed more than 6 months before the inclusion in the current study, in order to diminish possible interaction.

An increased WLR was found in the diabetic group with no retinopathy and in the group with proliferative retinopathy, when compared to healthy volunteers. This finding is consistent with previous studies that described arteriolar remodeling in subjects with diabetic retinopathy [15–17], as well as in subjects with prediabetes or in diabetic patients without signs of retinopathy [4, 18, 19]. Interestingly, no significant difference was demonstrated between the control and the NPDR group, nor in between the diabetic groups. It is worth mentioning that a consistent number of NPDR and PDR subjects previously benefited of antivascular endothelial growth factor (VEGF) injections for diabetic macular oedema. Anti-VEGF therapy has a vasoconstrictive effect [20] and could have determined the narrowing of retinal vessels. In diabetes, the artery remodeling process is due to smooth muscle cells hypertrophy and fibrosis [21], and it is best mirrored by WLR [22, 23]. The retinal blood vessels are offering direct information on the systemic microcirculation. Thus, retinal structural and functional vascular changes reflect the negative effects of hyperglycemia on the systemic microcirculation. In addition to this, the strong negative correlation in the study groups between the visual acuity and wall thickness and wall to lumen ration, respectively, might reflect how *in vivo* retinal parameters interfere with the visual function. These retinal arteriolar changes highlight underlying structural and functional alterations resulting from pathophysiological mechanisms involved in diabetes [24].

The analysis of the OCT-A parameters demonstrated significant differences in the superficial capillary plexus density between the groups. Two exceptions were recorded: control versus no-DR group and NPDR versus PDR group. It has been previously shown that after 3 months of anti-VEGF intravitreal injections, administered in order to control the diabetic macular oedema, some small vessels disappear on fluorescein angiography [20]. The occluded vessels might remain closed after anti-VEGF discontinuation. This might be the reason why SPD parameter was significantly higher in patients that had no anti-VEGF injections when compared to the group that received this treatment at least 6 months prior to the study.

OCT-A is a useful noninvasive imaging tool in the diagnosis and monitoring of retinal changes in diabetes [25]. Previous studies have identified a decrease in the parafoveal superficial and deep retinal vessel densities, with associated FAZ area increase in diabetic patients with [26, 27] or without retinopathy [28, 29], when compared to normal subjects. These changes suggest abnormal autoregulation of the retinal microcirculation in diabetic patients that can be tracked before the appearance of clinical findings.

We had expected to find consistent significant differences between same groups for both AAO and OCT-A studied vascular parameters, but these were only noticed when comparing healthy subjects with diabetic patients with proliferative retinopathy. In order to further clarify possible additional correlations, supplementary research is recommended. Larger sample sizes are needed in order to remove any source of bias. Separate analysis should be conducted for every stage of NPDR and PDR in order to draw a comprehensive conclusion. Moreover, several types of artefacts might have influenced the AO ophthalmoscopy and OCT-A analysis.

In conclusion, the present study tried to detect potential differences in the retinal microvasculature parameters for different diabetic retinopathy stages. AO retinal camera and OCT-A were able to detect microvascular changes between healthy subjects and diabetic patients. The results might reflect the superimposed effect of previous anti-VEGF treatment on the actual vascular status in both NPDR and PDR groups. Therefore, AO ophthalmoscopy and OCT-A are complementary techniques that are able to provide useful information about the topological and geometrical features of the retinal microvasculature from early onset of diabetic disease, thus having a promising role in the future. They might be used for routine follow-up, thus providing valuable prognostic information concerning the evolution of diabetic retinopathy.

## Figures and Tables

**Table 1:** Characteristics of groups included in the study (expressed as mean ± standard deviation or as median, unless otherwise indicated).

	Control	No DR group	NPDR group	PDR group
*N*	17	19	17	21
Sex (male/female)	8/9	7/12	11/6	18/3
Laterality (right/left)	14/3	8/11	15/2	11/10
Age (years)	44.82 ± 12.85	53.68 ± 20.16	56.24 ± 10.84	55.71 ± 13.06
BCVA (decimal)	1	1	0.8	0.8
Diabetes duration (years)	0	9.18 ± 6.70	13.94 ± 8.97	15.21 ± 10.71
Axial length (mm)	23.87 ± 1.08	23.70 ± 0.88	23.18 ± 0.60	23.47 ± 0.84
Diabetic maculopathy (number of subjects)	0	1	11	9

Legend: BCVA: best corrected visual acuity; DR: diabetic retinopathy; NPDR: nonproliferative diabetic retinopathy; PDR: proliferative diabetic retinopathy.

**Table 2:** Vascular parameters measured using both adaptive optics ophthalmoscopy and optical coherence ophthalmoscopy angiography in the four study groups (mean ± standard deviation).

Studied parameters	Control	No DR group	NPDR group	PDR group
Vessel diameter (*μ*m)	93.66 ± 14.06	94.30 ± 22.21	111.78 ± 30.21	97.16 ± 26.66
Lumen diameter (*μ*m)	75.70 ± 11.64	73.12 ± 19.52	88.73 ± 30.45	73.85 ± 24.95
Wall thickness (*μ*m)	9.20 ± 1.69	10.55 ± 2.12	11.25 ± 3.00	11.63 ± 2.90
Wall to lumen ratio	0.24 ± 0.046	0.30 ± 0.073	0.29 ± 0.11	0.34 ± 0.10
Wall cross sectional area (*μ*m^2^)	2499.05 ± 670.53	2878.81 ± 1143.75	3633.02 ± 1443.40	3194.96 ± 1371.45
Foveal avascular zone (mm^2^)	0.326 ± 0.133	0.360 ± 0.258	0.458 ± 0.143	0.402 ± 0.179
Superficial capillary plexus density	34.18 ± 1.40	33.85 ± 1.40	32.27 ± 1.93	32.31 ± 1.61

Legend: DR: diabetic retinopathy; NPDR: nonproliferative diabetic retinopathy; PDR: proliferative diabetic retinopathy.

**Table 3:** Results of the post hoc analysis between the four groups included in the study.

Studied parameters	Control ^∗^ no DR group	Control ^∗^ NPDR group	Control ^∗^ PDR group	No DR group ^∗^ NPDR group	No DR group ^∗^ PDR group	NPDR group ^∗^ PDR group
Vessel diameter (Games-Howell)	1.00	0.143	0.955	0.143	0.982	0.413
Lumen diameter (Games-Howell)	0.961	0.376	0.990	0.961	0.292	0.381
Wall thickness (Tukey)	0.372	0.90	0.20	0.844	0.530	0.964
Wall to lumen ratio (Games-Howell)	**0.029**	0.462	**0.005**	0.968	0.577	0.483
Wall cross sectional area (Tukey)	0.781	0.0381	0.297	0.248	0.841	0.682
Foveal avascular zone (Tukey)	0.954	0.173	0.630	0.445	0.915	0.824
Superficial capillary density (Tukey)	0.938	**0.006**	**0.007**	**0.038**	**0.046**	1

Legend: DR: diabetic retinopathy; NPDR: nonproliferative diabetic retinopathy; PDR: proliferative diabetic retinopathy.

**Table 4:** Results of the post hoc analysis between the four groups included in the study controlling for age.

Studied parameters	Control ^∗^ no DR group	Control ^∗^ NPDR group	Control ^∗^ PDR group	No DR group ^∗^ NPDR group	No DR group ^∗^ PDR group	NPDR group ^∗^ PDR group
Vessel diameter	1.000	0.256	1.00	0.215	1.000	0.420
Lumen diameter	1.000	0.575	1.00	0.261	1.000	0.300
Wall thickness	1.000	0.360	0.90	1.000	1.000	1.000
Wall to lumen ratio	0.501	1.000	**0.022**	1.000	1.000	0.120
Wall cross sectional area	1.000	0.111	0.872	1.000	0.445	1.000
Foveal avascular zone	1.000	0.833	1.000	1.000	1.000	1.000
Superficial capillary density	1.000	**0.015**	**0.016**	0.058	0.066	1.000

Legend: DR: diabetic retinopathy; NPDR: nonproliferative diabetic retinopathy; PDR: proliferative diabetic retinopathy.

**Table 5:** Results of Spearman's rank-order correlation between BCVA and the AO vascular and OCT-A parameters in the diabetic groups.

Spearmen correlation	Vessel diameter	Lumen diameter	Wall thickness	Wall to lumen ratio	Wall cross sectional area	Foveal avascular zone	Superficial capillary density
Coefficient (*r*_*s*_(55))	0.099	0.183	-0.367	-0.438	-0.052	-0.20	0.223
*p* value	0.463	0.174	**0.005**	**0.001**	0.701	0.894	0.133

## Data Availability

Raw and derived data supporting the findings of this study are available from the corresponding author [IEC] on request.
